# Long non-coding RNA *ROR* decoys gene-specific histone methylation to promote tumorigenesis

**DOI:** 10.1186/s13059-015-0705-2

**Published:** 2015-07-14

**Authors:** Jiayan Fan, Yue Xing, Xuyang Wen, Renbin Jia, Hongyan Ni, Jie He, Xia Ding, Hui Pan, Guanxiang Qian, Shengfang Ge, Andrew R. Hoffman, He Zhang, Xianqun Fan

**Affiliations:** Department of Ophthalmology, Ninth People’s Hospital, Shanghai JiaoTong University School of Medicine, Shanghai, 200025 P. R. China; Department of Biochemistry and Molecular Biology, Ninth People’s Hospital, Shanghai JiaoTong University School of Medicine, Shanghai, P. R. China; VA Palo Alto Health Care System, Stanford University Medical School, Palo Alto, CA 94304 USA

## Abstract

**Background:**

Long non-coding RNAs (lncRNAs) are not translated into proteins and were initially considered to be part of the ‘dark matter’ of the genome. Recently, it has been shown that lncRNAs play a role in the recruitment of chromatin modifying complexes and can influence gene expression. However, it is unknown if lncRNAs function in a similar way in cancer.

**Results:**

Here, we show that the lncRNA *ROR* occupies and activates the *TESC* promoter by repelling the histone G9A methyltransferase and promoting the release of histone H3K9 methylation. Suppression of *ROR* in tumors results in silencing of *TESC* expression, and G9A-mediated histone H3K9 methylation in the *TESC* promoter is restored, which significantly reduces tumor growth and metastasis. Without *ROR* silencing, *TESC* knockdown presents consistent and significant reductions in tumor progression.

**Conclusions:**

Our results reveal a novel mechanism by which *ROR* may serve as a decoy oncoRNA that blocks binding surfaces, preventing the recruitment of histone modifying enzymes, thereby specifying a new pattern of histone modifications that promote tumorigenesis.

**Electronic supplementary material:**

The online version of this article (doi:10.1186/s13059-015-0705-2) contains supplementary material, which is available to authorized users.

## Background

Long non-coding RNAs (lncRNAs) do not code for proteins and were previously considered ‘transcriptional noise’ [1–3]. Emerging studies have unraveled their important divergent cellular roles in epigenetic regulatory networks [4, 5]. For example, the lncRNA *Kcnq1ot1* can independently form chromatin loops to control genomic imprinting [6]. Increased interest has led to a greater number of studies focused on establishing paradigms for discovering new lncRNA functions.

Although only a small number of functional lncRNAs have been well characterized to date, these lncRNAs have been shown to control every level of the gene expression program [7], and a series of studies have further revealed that lncRNAs accomplish their functional roles by recruiting regulatory protein complexes to drive gene regulation [8, 9]. For instance, *ANRIL* mediates gene silencing by interaction and recruitment of CBX7, a component of the PRC1 complexes [10]; and *MEG3* lncRNA also recruits JARID2, an essential regulatory component of PRC2, to silence target genes during embryonic stem cell differentiation [11]. In theory, lncRNAs have the potential to modulate gene expression by repelling polycomb complexes away from chromatin.

As an important nucleotide molecule, single lncRNAs have always shown multiple roles in different organisms [12, 13]. *HOTAIR* is a classic lncRNA, and it has been found to promote cancer metastasis [14] and serves as a modular scaffold of histone modification complexes, thereby specifying its target gene [15]. Similarly, *MALAT1* interacts with SR splicing factors to control complex processes, such as the invasion of trophoblasts into the uterine wall [16], synaptogenesis [17], and tumor metastasis [18]. Recently, human LncRNA *ROR*, at only 2.6 kb in length, has been shown to reprogram differentiated cells to induced pluripotent stem cells (iPSCs) by directly targeted *OCT4*, *SOX2*, and *NANOG* through co-localization of the three factors close to its promoter region [19]. *ROR* is also involved in various key roles in DNA damage [20] and stem cell self-renewal [21]. However, whether *ROR* lncRNA has unidentified novel functional roles, especially in tumorigenesis, still remain unclear.

In this manuscript, we have attempted to identify the potential role of *ROR* lncRNA in the regulation of tumor progression. Using epigenetic approaches, we demonstrate that *ROR* lncRNA acts as a necessary decoy oncoRNA that plays an important regulatory role in tumorigenesis and represents a novel style of histone modification.

## Results

### Over-expression of *ROR* is significantly knocked down in tumors

**Fig. 1:**
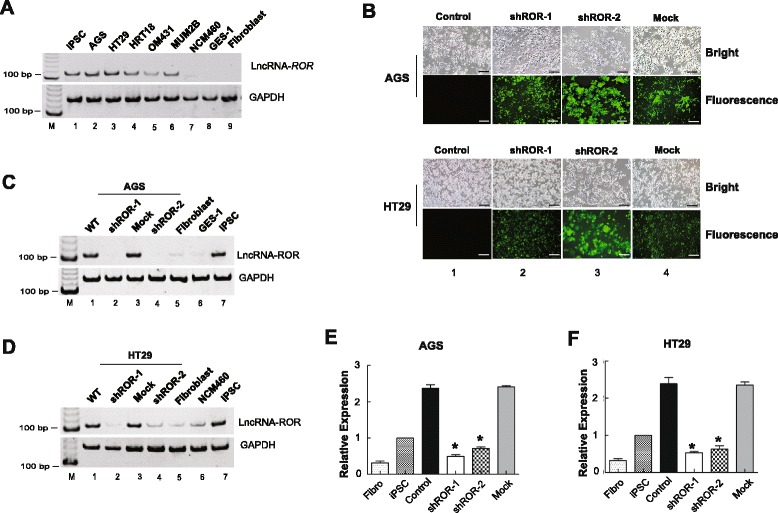
Knockdown of *ROR* expression in tumors. **a** *ROR* expression in different tumors and normal cells was measured by RT-PCR. *ROR* presented higher expression in a series of tumor cells than in two normal gastrointestinal cells (NCM460 and GES-1) and normal fibroblasts. iPSC cells were used as a positive control. *GAPDH* was used as the internal control. **b** *ROR* knockdown by two shRNAs. EGFP was used to track the expression of *ROR* shRNAs in AGS and HT29 cells. Scale bars: 100 μm. **c**-**f** RT-PCR (c, d) and real-time PCR (**e**, **f**) showed *ROR* expression in AGS and HT29 cells. M: marker; mock: empty pGIPZ vector, *P <0.05: compared with the control

To investigate our hypothesis, we first examined the expression of *ROR* in different tumor cells. Since the over-expression of *ROR* has been reported in iPSCs but not in human fibroblast cells [19], we selected these two cell types as positive and negative controls of *ROR* expression, respectively. Moreover, we also used normal intestinal mucosal cells (NCM460) and normal gastric epithelial cells (GES-1) to serve as controls for our tests of gastrointestinal tumor cells. As expected, we found that the expression of *ROR* was significantly increased in a series of tumor cells (Fig. 1a, lanes 2-6), whereas all of the negative controls remained weakly expressed (Fig. 1a, lanes 7-9). Thus, to decipher the potential role of *ROR* in tumors, we aimed to knockdown the expression of *ROR* using conventional RNAi methodology. Although a validated siRNA (siROR-1) of *ROR* lncRNA has been demonstrated [19], we designed another three siRNAs (siROR-2, siROR-3, and siROR-4) to search for the most efficient siRNA. However, we found that siRNA-1 was the most valuable siRNA with the ability to silence the expression of *ROR* in AGS (gastric cancer) and HT29 (colon cancer) tumor cells (Additional file 1: Figure S1). To exclude off-target effects, we next chose two siRNAs (siROR-1 and siROR-2) for the construction of the pGIPZ *ROR*-shRNA plasmids. The pGIPZ *ROR*-shRNA vectors with an EGFP marker were then packaged into lentiviruses and transduced into human AGS and HT29 cells. We used two control cell lines: one with a mock virus carrying the empty vector and one without the virus. Using EGFP as a tracking marker, we observed green fluorescence in AGS and HT29 cells (Fig. 1b).

We then detected the expression of *ROR* in stable cell clones selected with puromycin and found that *ROR* expression was knocked down in two shROR-expressing AGS (Fig. 1c, lanes 2 and 4) and HT29 tumor cells (Fig. 1d, lanes 2 and 4). We also further confirmed *ROR* expression by real-time PCR (Figs. 1e and f).

### *ROR* lncRNA contributes to tumor progression

**Fig. 2:**
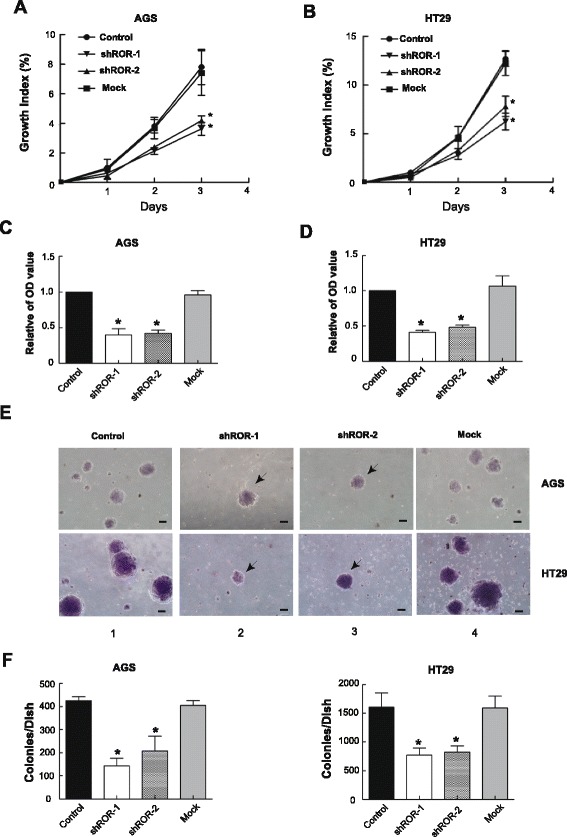
*ROR* modulates tumor growth and metastasis. **a**, **b** MTT assay showing tumor cell growth after *ROR* knock-down. Cell growth was obviously restrained at day 3 in *ROR*-silenced AGS and HT29 cells. The absorbance values were detected at 24 h, 48 h, and 72 h, the control was arbitrarily set at 100 % on day 1. **c**, **d** The migratory ability of *ROR*-silenced tumor cells. The ability to metastasize in *ROR*-silenced tumor cells was remarkably reduced compared with the untreated control cells. The 570 nm absorbance values of the control were set at 1. The migration detection was conducted at 24 h for AGS cells (c) and 48 h for HT29 cells (d). All of the data are presented as the mean ± SD. *P <0.05: compared with the control and mock. **e** Images of a soft agar tumor colony. Few visible colonies were observed in the *ROR* knockdown tumor cells. Bars: 100 μm. **f** Colony count statistics demonstrate tumor formation ability. Colony count statistics showed a significant reduction in *ROR*-silenced AGS and HT29 cells. Colony numbers were determined from three independent soft agar plates. All of the data are presented as the mean ± SD. *P <0.05: compared with the control and mock

Whether the tumor characteristics were significantly altered after *ROR* knockdown was then investigated. In an MTT assay, tumor cell growth showed an approximately two-fold decrease at day 3 in all *ROR*-silenced AGS and HT29 cells (Fig. 2a and b, triangle and inverted triangle), whereas the control (Fig. 2a and b, circle) and mock (Fig. 2a and b, Square) cells retained higher cell viability.

As another important indicator of tumor activity, we examined cell migration after *ROR* knockdown. Using a classical 24-well transwell system equipped with 8 μm polycarbonate filters, we seeded *ROR*-deficient and control cells into the upper chambers of the transwells and observed the capacity of cells to pass through polycarbonate filters with an 8 μm pore size. To avoid inaccuracies related to tumor migration from interfering with reduced cell proliferation, after cell seeding, the early detected point was arranged at 24 h for AGS cells and 48 h for HT29 cells. We found that the migratory ability of AGS cells was remarkably reduced as compared with that of the untreated control cells (Fig. 2c). Similarly, in *ROR*-silenced HT29 cells, the migratory ability showed a significant decrease (Fig. 2d). Next, we examined the cells’ ability to form tumor colonies *in vitro* by a soft agar formation assay. As expected, only a limited number of visible colonies were observed in the *ROR* knockdown AGS cells (Fig. 2e, arrow labeled). Colony count statistics demonstrated that significant reduction ultimately occurred in the *ROR*-silenced AGS cells (Fig. 2f, left). In addition, we also observed remarkably decrease in colonies in the *ROR*-silenced HT29 cells (Fig. 2f, right). These data indicated that *ROR* lncRNA plays a regulatory role in tumor progression and may serve as a new oncoRNA.

### Genome-wide analysis reveals that *TESC* serves as a target of *ROR*

**Fig. 3:**
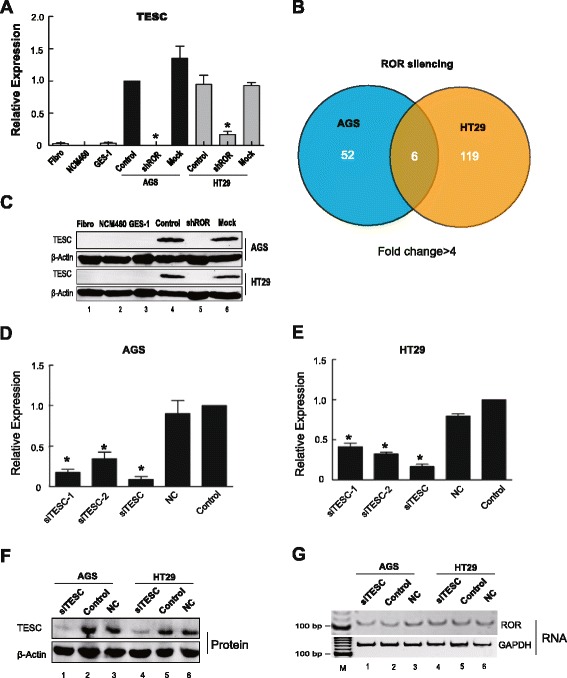
*TESC* serves as a target of *ROR*. **a** Real-time PCR measurement of mRNA expression of *TESC*. The expression levels of *TESC* in tumor cells were significantly higher than normal. Once *ROR* was silenced in tumor cells, there was a remarkable decrease in *TESC* expression. NCM460: normal colon cell; GES-1: normal gastric cell. **b** Overlapping altered genes from lncRNA-*ROR* knockdown cells from AGS and HT29 cells. By using four-fold changes as baseline, there were approximately 58 altered genes after depletion of *ROR* in AGS and 125 altered genes in HT29. A total of six genes were changed in AGS and HT29 cells after *ROR* knockdown. **c** Western blot showing that the expression of *TESC* in *ROR* silenced AGS and HT29 tumor cells. The expression of TESC was greatly decreased in *ROR*-depleted tumor cells as well as in normal cells, including fibroblasts, normal colon cell NCM460, and normal gastric cell GES-1. **d**, **e** Silenced expression of *TESC* using siRNAs in AGS (d) and HT29 cells (e). Real-time PCR demonstrated siTESC provided the optimal deletion of *TESC*. Experiments were performed 48 h following siTESC (125 pmol) and control siRNA(125 pmol) treatment. *P <0.05: compared with the control and NC. NC: non-silencing control. **f** Western blot demonstrating that siTESC efficiently silenced *TESC* at the protein expression level in AGS and HT29 cells. All experiments were performed 48 h following siTESC (125 pmol) and control siRNA (125 pmol) treatment. NC: non-silencing control. **g** RT-PCR showing that *ROR* expression was not significantly changed when *TESC* was silenced in AGS and HT29 cells

To determine the factors that coordinated these tumor variations after *ROR* knockdown, we examined gene expression by a genome-wide cDNA array. Compared with untreated tumor cells, the expression of at least 58 genes was significantly changed by more than four-fold (Additional file 2: Table S1, GEO accession number: GSE67416, fold >4), including genes that were both up- and downregulated in AGS cells. We then selected seven notable altered genes: *AKR1C1*, *AKR1C3*, *LMO4*, *MGST1*, *LXN*, *TIMP3*, and *TESC*. The results indicate that *TESC* was significantly decreased in *ROR*-depleted cells (Fig. 3a). Similarly, we also further confirmed the reduction of the other six candidate genes through real-time PCR (Additional file 3: Figure S2 a-f). Unfortunately, of these potential targets, altered tumor activity was not detected after silencing their expression by siRNA except for in *TESC* (Additional file 3: Figure S2 g, h). We also detected gene expression after *ROR* depletion in HT29 cells by genome-wide cDNA array. Intriguingly, in HT29 cells, *TESC* was also one of the most significantly altered genes (Additional file 4: Table S2, GEO accession number: GSE67416, fold >4), and its expression overlapped with that of AGS cells (Fig. 3b). Moreover, because the regulatory role for *TESC* in tumorigenesis was not indicated, we then explored whether *TESC* serves as a possible downstream *ROR*-targeting gene to modulate tumor activity.

To test this hypothesis, we compared TESC expression at the protein level between tumors and three negative controls: NCM460, GES-1, and fibroblasts. As expected, we found that TESC expression was significantly increased in AGS and HT29 tumor cells (Fig. 3c, lane 4) and not observed in the three negative control cells (Fig. 3c, lanes 1, 2, and 3). Once *ROR* was silenced, TESC showed only weak expression in the *ROR* knockdown cells (Fig. 3c, lane 5), which is consistent with the results of the cDNA array and real-time PCR. We then knocked down *TESC* using siRNA, and three siRNAs (siTESC, siTESC-1, and siTESC-2) were designed to test the efficiency of silencing. The results showed that siTESC was more efficient than the other two siRNAs in silencing *TESC* at the mRNA transcript level (Fig. 3d and e). We thus used siTESC to examine the TESC protein expression level. As expected, TESC was successfully silenced by siTESC (Fig. 3f, panel 1, lanes 1 and 4). Intriguingly, *ROR* expression was not significantly changed when *TESC* was silenced (Fig. 3g, panel 1, lanes 1 and 4). These data support our hypothesis that diminished *TESC* expression is triggered by lncRNA-*ROR* depletion and *TESC* acts as a regulatory target of *ROR* lncRNA.

### *ROR* modulates tumor activity through its downstream target *TESC* gene

**Fig. 4:**
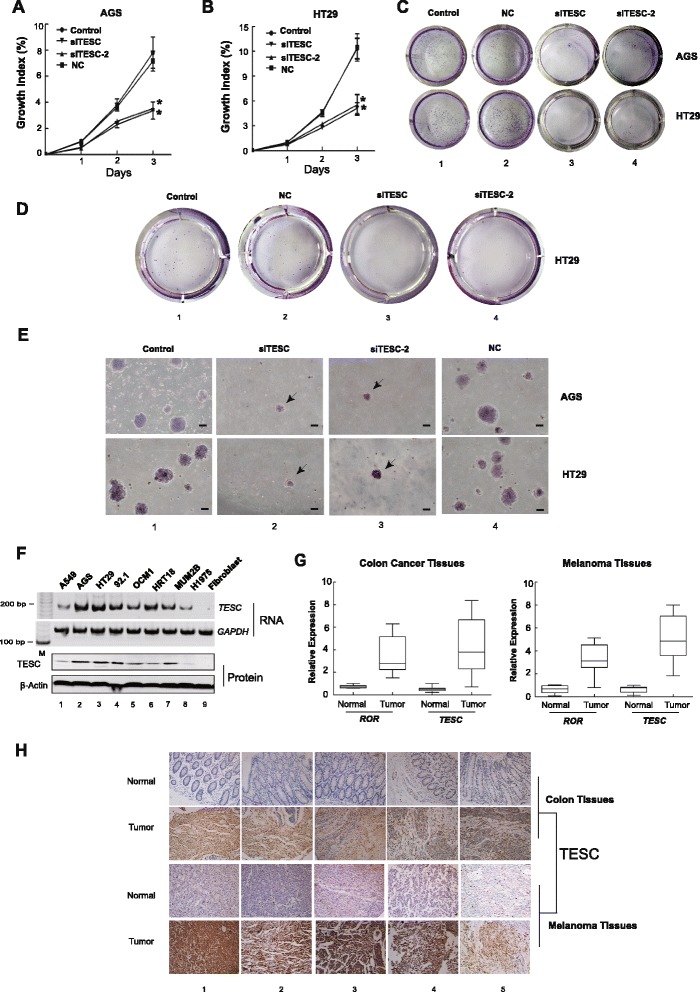
*TESC* is a potential novel oncogene. **a**, **b** Declining cell growth in AGS (a) and HT29 (b) cells after *TESC* silencing. The absorbance value of the controls at day 1 was arbitrarily set at 100 %. *P <0.05: compared with the control and NC. **c** Images of metastatic tumor cells in *TESC*-silenced cells. The migratory ability of two siTESCs-treated AGS and HT29 tumor cells were significantly decreased. The migration assay was conducted 48 h after transfection with siTESCs or control siRNA. **d**, **e** Soft agar colony assay showing the ability of tumor formation *in vitro*. A tiny colony observed in whole-well testing (d) and under the microscope (e). Bars: 100 μm. Arrow: tumor colony. **f** RT-PCR showing the high expression of *TESC* in a variety of tumor cells. *TESC* was abundant in tumor cells but not in fibroblasts. **g** *ROR* and *TESC* expressionl evels in normal tissues, colon cancer, and melanoma tissues. Both *TESC* and *ROR* were highly expressed in colon cancer and melanoma tissues compared with that of normal tissues. The expression of *TESC* was increased with the level of *ROR* in tumors. **h** Immunohistochemical staining of TESC in tumor and normal tissues. TESC expression in tumor sections from five colon cancer patients and five melanoma patients was higher than normal tissues (original magnification, 200×)

To exclude off-target effects, we then chose two validated siRNAs (siTESC and siTESC-2) for next experiments. After *TESC* silencing, we also found a near two-fold decline in the growth of AGS cells (Fig. 4a). In addition, *TESC* silenced-HT29 cells showed similarly decreased cell proliferation (Fig. 4b). To further define the role of *TESC* in tumor migration and formation, we first examined the ability of cells to migrate in *TESC*-silenced AGS and HT29 tumor cells. Following the above protocol for the transwell assay, we observed a significantly decreased metastasis rate after 48 h (Fig. 4c, lanes 3 and 4) compared with that of the controls (Fig. 4c, lanes 1 and 2). In a classical tumor formation assay *in vitro*, we also noticed tiny colonies in *TESC*-deficient cells through whole well testing (Fig. 4d, lanes 3 and 4) or under the microscope (Fig. 4e, lanes 2 and 3). These results provide direct evidence that *TESC* may be a newly proposed oncogene with functions in tumor growth and metastasis.

To determine the role of *TESC* as a potential novel onco-target gene in gastrointestinal tumor cells, we then examined whether aberrant *TESC* expression was enriched in other malignant tumor cells. In addition to the above confirmed gastric (Fig. 4f, lane 2) and colonic tumors (Fig. 4f, lanes 3, 6), we showed that abundant *TESC* was detected in 92.1, OCM1 and MUM2B cells, which are three malignant ocular melanoma cell lines (Fig. 4f, lanes 4-5, 7), but not in non-small cell lung cancer cells (Fig. 4f, lanes 1, 8). To verify the clinical significance of *TESC*, we collected a set of tumor tissues paired with adjacent normal tissue from diagnosed patients, including gastro tumor (n = 20) and ocular melanoma (n = 20). We then examined whether the expression of *TESC* was correlated with *ROR* in those tumor tissues. As expected, both *ROR* and *TESC* presented weak expression in normal samples. However, compared with the adjacent healthy specimens, a prominent increase in *TESC* expression was detected once *ROR* was significantly overexpressed, either in colon tumors or ocular melanomas (Fig. 4g). An immunohistochemistry staining assay was then performed to detect TESC protein expression in tumor tissues. The results clearly showed that TESC protein expression was remarkably increased in the tissues of gastrointestinal tumors (Fig. 4h, panel 2) and melanoma (Fig. 4h, pane 4) compared with that of adjacent normal tissues (Fig. 4h, panels 1 and 3). These data further highlight the clinical importance of *TESC* and *ROR* in gastrointestinal cancer and ocular melanoma.

### *ROR* abolishes histone H3K9 methylation of the *TESC* gene

**Fig. 5:**
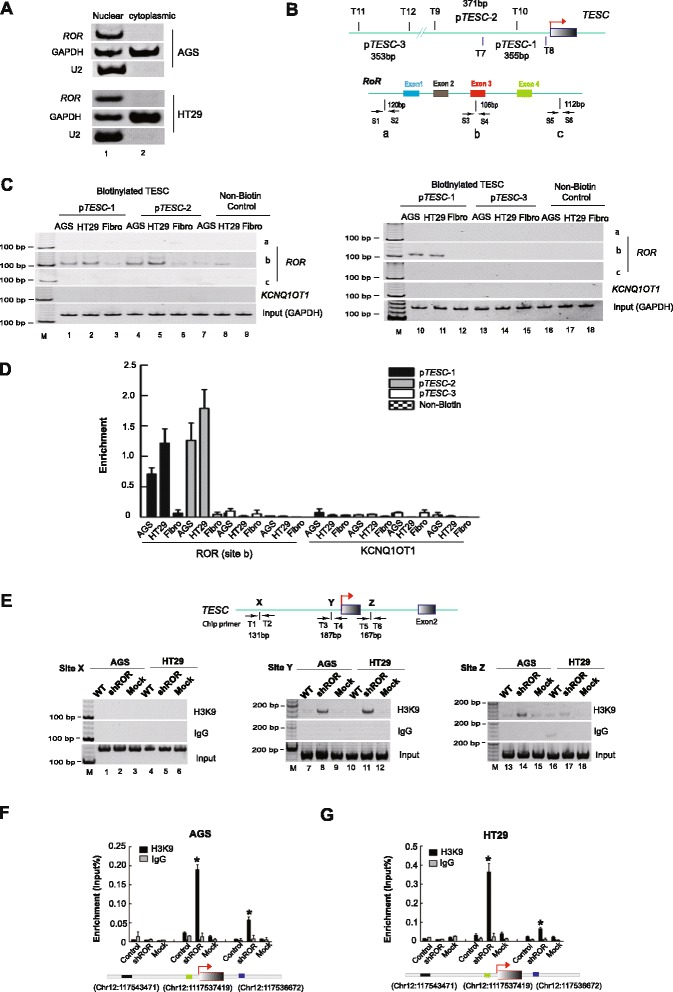
*ROR* abolishes histone H3K9 methylation of the *TESC* promoter. **a** The location of mature *ROR. ROR* was mainly present in the nucleus. *U2* RNA was used as a positive control for nuclear RNA. **b** Schematic diagram of the *TESC* promoter region and *ROR* lncRNA. T7 through T12 and S1 through S6: primer names, arrow: transcriptional direction, p*TESC*-1 and p*TESC*-2: two different biotinylated *TESC* promoter fragments; p*TESC*-3: biotinylated DNA fragments 10 kb upstream of *TESC*. Sites a, b, and c: different detecting locations of RNA IP. **c** The interaction of *ROR* and the *TESC* promoter. The *TESC* promoter (p*TESC*-1 and p*TESC*-2) specifically interacts with exon 3 of the *ROR* (site b). p*TESC*-3 was used as a negative control locus. Fibroblasts and lncRNA-*KCNQ1OT1* were used as negative controls. Input: total RNA was reverse transcribed before incubation with labeled p*TESC* fragments and amplified with *GAPDH* primers. **d** TaqMan real time-PCR used to quantify the enrichment of *ROR* on the *TESC* promoter. *TESC* binds near exon 3 of *ROR* (site b). p*TESC*-3 was used as a negative control locus. *KCNQ1OT1* was used a negative control. **e**-**g** ChIP assay detecting H3K9 trimethylation of the *TESC* promoter. Sites X, Y, and Z: different sites used in this assay. IgG: native control. All data are presented as the means ± SD. *P <0.05: compared with the control and mock

To determine the precise mechanism underlying *ROR* regulation of *TESC* expression in tumors, we examined the cellular location of the mature *ROR* transcript. It has been reported that the small nuclear RNA (snRNA) *U2* is common in the nucleus and participates in RNA splicing in the assembly and function of canonical spliceosomes [22]. Thus, *U2* snRNA was utilized as a positive reference for the examination of *ROR* location. By isolating both nuclear and cytoplasmic RNA, we showed that *ROR* was mainly present in the nucleus, at least in AGS (Fig. 5a, panel 1, lane 1) and HT29 cells (Fig. 5a, panel 4, lane 1). Thus, we investigated the possibility of *ROR* interacting with the *TESC* promoter. In a DNA pull-down assay, two biotin-labeled DNA fragments (p*TESC*-1 and p*TESC*-2) overlapping the *TESC* core promoter were used to incubate with total RNA. A control DNA fragment (p*TESC*-3) from 10 kb upstream of *TESC* was used as a negative control (Fig. 5b). After the pull-down and cDNA synthesis, we showed that the center of the *ROR* transcript, near exon 3 of *ROR*, could interact with the *TESC* promoter in *ROR*-expressing tumor cells (Fig. 5c, lanes 1-2 and 4-5). Fibroblasts (Fig. 5c, lanes 3 and 6) and non-biotin controls failed to show this DNA-RNA interaction (Fig. 5c, lanes 7-9). An interaction between *ROR* and a negative locus (p*TESC*-3) was not observed in AGS and HT29 cells (Fig. 5c, right, lanes 13-14). The non-biotin controls did not show an interaction too (Fig. 5c, lanes 16-18). To further validate the binding of *ROR* and *TESC* promoter, we performed a TaqMan qPCR analysis to quantitate the enrichment of *ROR* at the *TESC* promoter and showed that *ROR* was bound to the core promoter of *TESC* compared with that of the controls (Fig. 5d), suggesting that *ROR* may regulate *TESC* expression via chromatin-level machinery.

We then determined whether *ROR* binding of the *TESC* promoter could influence epigenetic modifications, such as histone methylation. Using a ChIP assay, we observed that H3K9 trimethylation of the *TESC* promoter was abolished in *ROR*-expressing AGS cells, which led to higher expression (Fig. 5e, lane 7). Once *ROR* was depleted, H3K9 trimethylation at the *TESC* promoter recovered dramatically, leading to *TESC* silencing (Fig. 5e, lanes 8 and 14). A similar observation was made in HT29 cells (Fig. 5e, lanes 10-12), and the above results were further confirmed by a quantitative ChIP-PCR assay (Fig. 5f and g). Moreover, we found that there was no significant global alteration in H3K9 trimethylation after *ROR* silencing (Additional file 5: Figure S3), indicating *ROR* itself was not able to regulate the activity of H3K9 methyltransferase and was likely to modulate H3K9 trimethylation of the *TESC* promoter through directing the binding of H3K9 methyltransferase to the target regions of genome. Because G9A is an H3K9 methyltransferase, it was evaluated to examine its interaction with *ROR*. As suggested, an RNA IP assay demonstrated that *ROR* failed to bind with the G9A protein, at least at sites a, b, and c (Additional file 6: Figure S4). These data raised the possibility that lncRNA-*ROR* may compete with the polycomb protein G9A in a manner other than recruitment to abolish H3K9 trimethylation.

### *ROR* competes with G9A methyltransferase *in vitro*

**Fig. 6:**
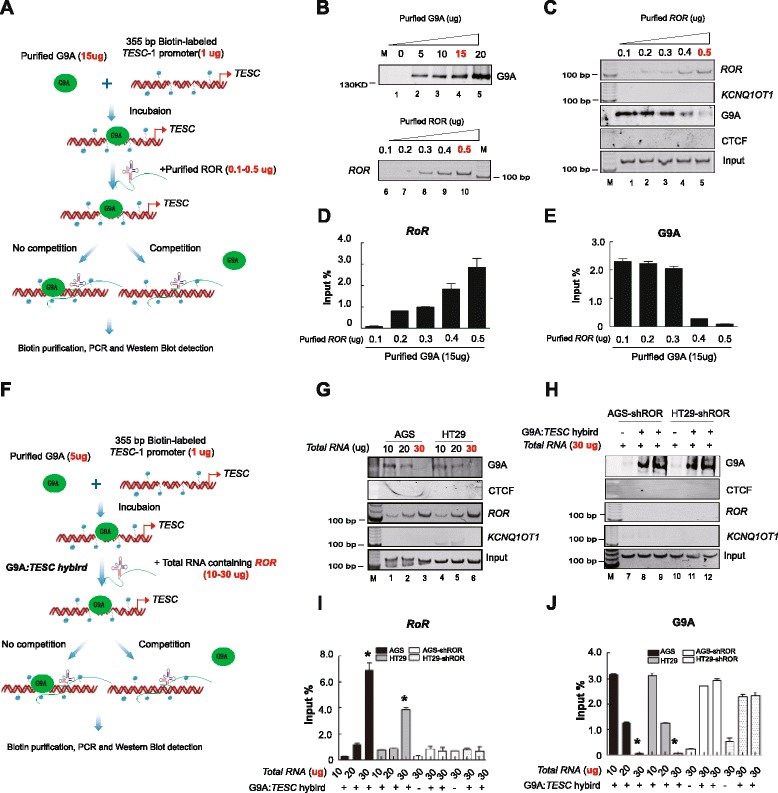
*ROR* competes with G9A at the *TESC* promoter *in vitro*. **a** Schematic diagram showing the first experimental design of purified *ROR* competing with purified G9A at the *TESC* promoter. We used 15 μg purified G9A protein and incubated it with 1 μg biotinylated *TESC*-1 DNA probe. After incubation, 0.1-0.5 μg purified *ROR* lncRNA was then added to the reaction mixture to compete with the G9A:*TESC*-*1* hybrid. PCR and western blot were used to detect the residual amount of *ROR* lncRNA and G9A protein after biotin purification and precipitation, respectively. Red helix: *TESC* DNA; green cycle: G9A protein; small blue cycle: biotin labeled; red arrow: transcriptional direction of *TESC*. **b** Expression of purified G9A and purified *ROR* lncRNA. M: maker. **c** Different amount of purified *ROR* competing with the G9A:*TESC*-*1* hybrid. In the presence of 0.4 μg purified *ROR*, G9A significantly reduced binding with the *TESC* DNA fragment. CTCF and *KCNQ1OT1* were used as a negative control. Input: collected biotinylated *TESC*-1 fragments before pull-down and PCR with primers aligned with the *TESC* promoter. **d**, **e** The quantitive assay of *RoR* and G9A interaction. The image J software was used to quantitate the RNA:protein interaction, *P <0.05. **f** Schematic diagram showing the modified experimental design of total RNA containing *ROR* competing with purified G9A at the *TESC* promoter. We used 5 μg purified G9A incubated with 1 μg biotinylated *TESC*-1 probe. After incubation, different amounts of total RNA extracted from tumor cells were then added to the reaction mixture to compete with the G9A:*TESC*-*1* hybrid. PCR and western blot were used to detect the amount of *ROR* lncRNA and G9A protein after biotin purification and precipitation, respectively. **g**, **h** Different amounts of total RNA containing *ROR* competed with the G9A:*TESC*-*1* hybrid. In the presence of 30 μg total RNA extracted from tumor cells, the G9A protein significantly reduced binding with the *TESC* DNA fragment (g). The total RNA of *ROR* silencing AGS and HT29 tumor cells failed to compete with the G9A:*TESC* hybrid (h). CTCF and *KCNQ1OT1* were used as a negative control. Input: collected biotinylated *TESC*-1 fragments before pull-down and PCR with primers aligned with the *TESC* promoter. **i**, **j** Competition of total RNA and G9A is shown in i and j. The RNA:protein interaction was quantitated by the image J software,*P <0.05

We next determined whether *ROR* lncRNA was capable of competing with G9A at its target DNA, the *TESC* promoter by conducting a competition assay with G9A and *ROR*. First, 355 bp biotin-labeled double stranded DNA fragment from the *TESC* promoter (p*TESC*-1) was synthesized, and then 1 μg p*TESC*-1 DNA probe was incubated with purified G9A protein to form a protein:DNA hybrid. To examine the ability of *ROR* lncRNA to compete with the G9A protein, we then added purified *ROR* lncRNA produced by *in vitro* RNA synthesis to the reaction mixture. If the *ROR* was a real competitor of G9A, *ROR* would occupy the binding site of G9A, and free G9A protein would be released from the *TESC* DNA. Following this route, after the biotin-streptavidin pull down, PCR or western blot was used to determine the residual amount of *ROR* and G9A, respectively (Fig. 6a). Before the assay, we evaluated the amount of purified *ROR* and purified G9A used in this examination. As shown in Fig. 6b, we found that 5-20 μg purified G9A protein was sufficient for detection by western blot (Fig. 6b, lanes 2, 3, 4 and 5). We also showed that 0.3-0.5 μg purified *ROR* could be successfully detected via PCR assay (Fig. 6b, lanes 8, 9, and 10).

Based on the above amount of purified RNA and protein, 15 μg purified G9A protein was used in this competition assay to form the G9A protein:*TESC* DNA hybrid. After adding the variant amount of purified *ROR*, we found that *ROR* lncRNA significantly abolished the binding of G9A with the *TESC* DNA fragment (Fig. 6c, panel 3, lane 5) in the presence of 0.5 μg purified *ROR* (Fig. 6c, panel 1, lane 5). The negative controls *KCNQ1OT* 1 lncRNA (Fig. 6c, panel 2) and CTCF protein (Fig. 6c, panel 4) did not show expression in this assay. We also confirmed the results in quantitive assay (Fig. 6d and e).

Next, we modified the experimental design to determine whether the *ROR* lncRNA in AGS and HT29 cells could compete with the G9A protein *in vitro*. In the revised protocol, the amount of purified G9A used in experiment was reduced to 5 μg, and total RNA extracted from AGS and HT29 cells was used instead of purified *ROR* RNA (Fig. 6f). Following *in vitro* incubation and purification, we demonstrated that 30 μg total cellular RNA containing *ROR* lncRNA (Fig. 6g, panel 3, lanes 3 and 6) successfully abrogated the binding of G9A protein to the *TESC* promoter in AGS and HT29 cells (Fig. 6g, panel 1, lanes 3 and 6).

We then determined whether *ROR* depletion in AGS and HT29 cells could influence the competition of RNA and protein. In accordance with the protocol shown in Fig. 6f, 30 μg total RNA from *ROR*-deficient AGS and HT29 cells were used to compete with the G9A:*TESC* hybrid. As expected, after *ROR* silencing (Fig. 6h, panel 3), G9A protein was maintained to interact with p*TESC* for forming G9A:*TESC* hybrid (Fig. 6h, panel 1, lanes 8, 9, 11, and 12) as compared with non-hybrid controls (Fig. 6H, panel 1, lanes 7 and 10). Similarly, we also detected consistent results in quantitive assay (Fig. 6i and j), and the results demonstrated that *ROR* lncRNA acted as a *bona fide* competitor of the G9A protein.

### *ROR* repels the endogenous G9A methyltransferase

**Fig. 7:**
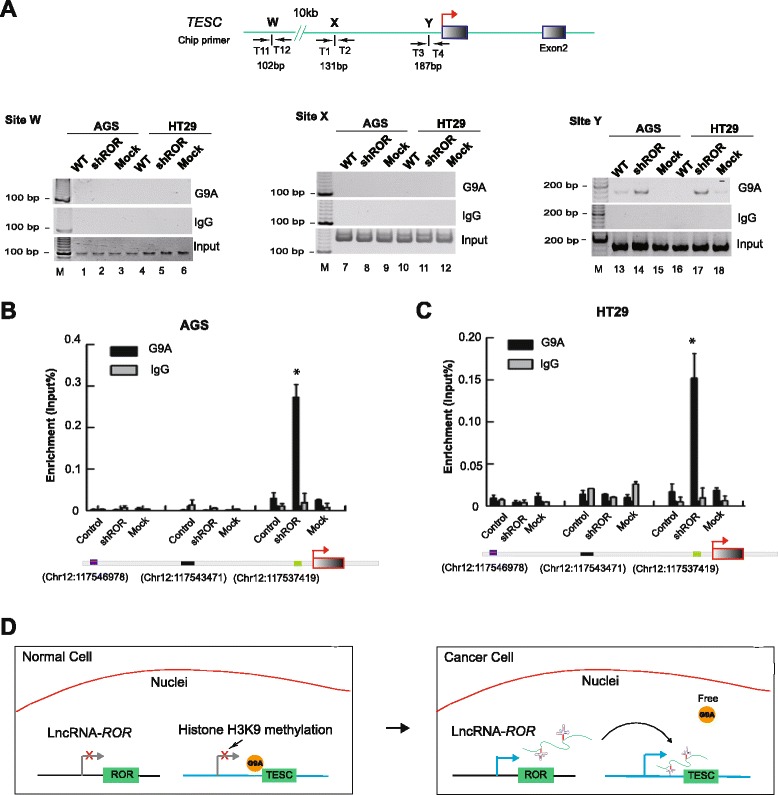
*ROR* repels endogenous G9A methyltransferase. **a** ChIP assay demonstrating that *ROR* blocked the recruitment of G9A to the *TESC* promoter. IgG is used as a native control. T1 through T4 and T11 through T12: primer names; arrow: transcriptional direction; sites X, Y and W: ChIP detecting sites; grey box: exons of *TESC* genes. **b**, **c **The qPCR-ChIP assay showing the interaction of G9A with the *TESC* promoter. The detection of ChIP sites on the chromosome is listed at the bottom. IgG: native control. All of the data are presented as the mean ± SD. *P <0.05: compared with the control and mock. **d** Decoy model of *ROR* regulation in tumorigenesis. In normal cells, *ROR* lncRNA was silenced and the G9A methyltransferase could freely modify the *TESC* promoter and provide histone H3K9 methylation to depress *TESC* expression; however, in cancer cells, *ROR* was abnormally activated and blocked the G9A interaction with the *TESC* promoter, and free G9A failed to methylate the *TESC* promoter and induced aberrant *TESC* expression, triggering tumorigenesis

To determine whether *ROR* abolished G9A binding at the *TESC* promoter in the cell nucleus, we used a ChIP assay and showed that *ROR* blocked the recruitment of G9A to the *TESC* promoter (Fig. 7a, lane 13, 16) in the two controls (mock and non-treatment cells); however, with *ROR* silencing, G9A could dramatically bind to the *TESC* promoter nearby (Fig. 7a, lane 14, 17). We used two negative ChIP sites (X and W) to exclude a non-specific interaction. As expected, G9A binding was not measured with the *TESC* promoter regardless of the *ROR* expression status at these native sites.

Similarly, the ChIP-QPCR data were consistent with this finding as well (Fig. 7b and c), and the results demonstrate that lncRNA-*ROR* repels the polycomb core protein G9A away from the *TESC* promoter.

## Discussion

An intriguing common theme has emerged wherein large ncRNAs mediate epigenetic mechanisms by forming ribonucleic–protein complexes that impart key regulatory functions in cellular circuits [23–27]. Most of the well-defined lncRNAs, such as *HOTAIR*, lncRNA-*p21*, *ANRIL*, and *MALAT*-1, share a common functionality in the formation of RNA–protein complexes with chromatin regulatory factors [1]. However, this study provides new insight into the lncRNA-specifying mechanism underlying gene-specific histone modification of tumorigenesis and shows that *ROR* acts as a decoy oncoRNA to block the recruitment of chromatin regulatory factors (G9A methyltransferase), abolish histone H3K9 modification of the *TESC* promoter and induce abnormal tumor growth and metastasis. Without *ROR* expression, the G9A protein is restored to the *TESC* promoter, thereby silencing *TESC* expression by targeted histone H3K9 methylation and leading to significantly depressed tumor progression (Fig. 7d).

For decades, the classic cause of tumorigenesis was assumed to be alterations in the balance of gene expression that maintains cellular homeostasis [28]. However, recent studies have suggested that an expanded definition beyond protein-coding genes must also include lncRNAs [16, 29]. A number of profiling and characterization studies identified critical roles for long ncRNAs in eventual cancer metastasis and development. For example, *p53* induces lncRNA-p21, which in turn represses numerous genes globally by recruiting the repressor protein hnRNP-K to modulate tumor progression [30], suggesting that lncRNAs may act as key regulatory nodes in multiple transcriptional pathways and serve as a signal or a convenient regulator to track the transcriptional activity of a target gene during cancer development. In support of this hypothesis, we demonstrate that *ROR* lncRNA serves as a regulatory hinge in a transcriptional framework to spread a tumorigenic signal to the downstream target *TESC* and consequently initiate tumor development by activating *TESC* expression. As a central node of the regulatory network, the characterization of *ROR* may represent a new and rapid indicator without requiring additional translational processes, which may accelerate its diagnostic use in clinics.

It should be noted that the *TESC* gene (*Tescalcin*) was originally identified from the early stages of gonadal differentiation in mouse testis [31]. The *TESC* gene encodes a 24 KDa EF-hand calcium-binding protein [32] and has been proposed as a novel genetic influence on hippocampal size and potential risk factor for cognitive decline and dementia that causes Alzheimer’s disease [33]. Thus far, evidence does not indicate the regulatory role of the *TESC* gene in tumors. In this report, however, we clearly demonstrate that *TESC* contributes to tumor growth and metastasis and is considered a novel oncogene in tumor development.

Although we cannot theoretically eliminate other genetic or epigenetic origins in *TESC* expression, this is the first study to imply that *TESC* expression is highly dependent on histone H3K9 methylation of its promoter locus. Thus, it would be of great interest to focus on the identification of other factors to better understand *TESC* regulation. It also should be noted that *ROR* itself did not directly contribute to the activity of histone H3K9 methytransferase for altering global H3K9 methylation. Alternatively, nuclear and chromatin-associated *ROR* mediated the H3K9me3 marks in its target loci by coordinating the recruitment of G9A methytransferase. Further studies are required to validate the profile of *ROR* occupancy along the whole genome for exploring the role of more *ROR*-targeting epigenetic marks in tumorigenesis.

Although it is not surprising that epigenetically directed gene expression affects tumor progression by abolishing core modifying complexes with a decoy protein [34] or by capturing miRNA with competing endogenous RNAs (ceRNAs) [35], a nuclear decoy lncRNA that integrates and conveys contextual cues for tumor progression was observed here rather than a decoy protein or miRNA sponge. *Tsix* and *Jpx* ncRNAs have also been proposed as decoys for PRC2 recruitment [36] or CTCF binding [37] in X-chromosome inactivation (XCI). Nevertheless, to our knowledge, this is the first example where lncRNA serves as a decoy oncoRNA for the modulation of tumorigenesis, and it provides an alternative strategy for exploring potential mechanisms underlying tumorigenesis in other cancers.

## Conclusions

In summary, our results reveal a completely novel mechanism in which *ROR* lncRNA serves as a decoy oncoRNA and specifies a new pattern of histone modifications in tumorigenesis. Since a number of lncRNAs are aberrantly expressed in a variety of diseases, it is possible that those lncRNAs may play regulatory roles through a similar decoy pattern rather than through the recruitment of polycomb protein complexes to their targets, thereby providing new avenues for the exploration of lncRNA biology and providing potential targets for the diagnosis and treatment of disease.

## Materials and methods

### shRNA-expressing plasmid construction

The two shRNA sequences (shROR-1: CCTGAGAGTTGGCATGAAT; shROR-2: GGTTAAAGACACAGGGGAA) that targets *ROR* were obtained by PCR with X*ho* I-M*lu* I sites and then cloned into the X*ho* I- M*lu* I sites in the pGIPZ lentivirus vector (System Biosciences, USA).

### RNA extraction and reverse transcription-PCR analysis

Total RNA was extracted using TRI-REAGENT (Invitrogen, USA) according to the manufacturer’s instructions, and cDNA was synthesized using the PrimeScript RT reagent kit (Takara, Japan). A PCR analysis was performed using KlenTaq I mix, and amplified PCR products were quantified and normalized using *GAPDH* as a control. The PCR cycle parameters for *ROR* and *TESC* expression were as follows: 33 cycles of denaturation at 95 °C for 30 s, optimal annealing temperature for 30 s, extension at 72 °C for 30 s and a final extension at 72 °C for 5 min.

### Lentivirus package

The 293 T cells were cultured in Dulbecco’s modified Eagle’s medium (Gibco, USA) supplemented with 10 % (vol/vol) fetal bovine serum and maintained at 37 °C at a concentration of 6,000,000 cells and transfected using Lipofectamine 2000 reagent (Invitrogen) with 3 μg GIPZ-shROR, 3 μg pMD2.D, and 6.0 μg PsPax. After incubation overnight with 293 T cells, the media was replaced with 5 mL fresh medium. The virus-containing supernatants were collected at 48 h and 72 h after transfection and then mixed and filtered through a 0.45 μm cellulose acetate filter (Sartorius). The viral supernatants were concentrated with Amicon Ultra-15 Centrifugal Filter Units (Millipore, USA) at 4 °C and spun at 5,000 rpm for 30 min. The colonies with GFP expression were selected for subsequent culture after incubation with 4 g/mL puromycin for 2 weeks.

### MTT assay

Cells were seeded at 5,000 cells per well in flat-bottomed 96-well plates. At the end of the incubation time, 20 μL 5 mg/ml 3-(4, 5-dimethylthiazol-2-yl)-2, 5-diphenyl-2H-tetrazolium bromide (MTT) (Sigma-Aldrich, USA) in phosphate-buffered saline (PBS) was added to each well. After 3 h, the media was discarded and the cells were lysed with 100 μL dimethylsulfoxide. The cells were incubated for a further 30 min at 37 °C with gentle shaking. The optical density was determined with a microplate reader at 570 nm. The absorbance values were normalized to the values of the 0 h tumor cells, and the 24 h wild-type tumor cells were set to 100 % to calculate the percentage of viable cells.

### Migratory ability assay

The migratory ability of the *ROR*-depleted cells was evaluated using a 24-well Transwell system (Corning, USA) equipped with 8 μm pore size polycarbonate filters according to the manufacturer’s instructions. The upper compartment contained 5 × 10^5^ cells that were seeded into the upper chambers of the Transwell system and supplemented with 1 % fetal bovine serum. The lower compartment contained 15 % fetal bovine serum, was fixed with 100 % methanol and stained with 0.1 % crystal violet before photographing. The crystal violet was washed from the migrated cells using 100 μL 33 % acetic acid. The absorbance of the washed down liquid was determined with a microplate reader at 570 nm.

To measure the migratory ability of cells after *TESC* knockdown, cells were seeded at 2 × 10^5^ cells per well in 6-well plates,transfected using Lipofectamine 2000 (Invitrogen) in Opti-MEM I Reduced Serum Medium (Invitrogen) with 125 pmol siRNA, and after 6 h, they were incubated in 10 % fetal bovine serum. At 24 h post-transfection, the cells were harvested in trypsin, and 5 × 10^5^ cells were seeded into the upper chambers of the Transwell system and supplemented with 1 % fetal bovine fresh medium. The lower compartment contained 15 % fetal bovine serum, andcells werecultured at 37 °C and 5 % CO_2_ for another 24 h. After 48 h post-transfection, the lower compartment was fixed with 100 % methanol and stained with 0.1 % crystal violet before photographing.

### Soft agar tumor formation assay

A soft agar colony formation assay was performed in 6-well plates. One milliliter of the bottom layerconsisted of 0.6 % agar in complete medium, and it was spread in each of the 6-well plates. A total of 20,000 cells were suspended in 1.0 mL of complete medium containing 0.3 % agar and seeded into each well. The cultures were fed every 3 to 4 days with 300 μL of complete medium for 3 to 4 weeks. For quantification, the colonies grown in soft agar were stained with 0.005 % crystal violet. The size of the colonies was determined using Adobe Photoshop.

### Genome-wide cDNA array

Total RNA was prepared from AGS, knockdown *ROR* of AGS and AGS-mock cells using an RNeasy Mini Kit (Qiagen, USA). cDNAs were amplified and labeled using a Quick Amp Labeling Kit (Agilent Technologies, USA) and hybridized onto an Agilent oligomicroarray. Statistical analyses and data normalization were conducted using the Genespring GX software (Agilent Technologies). Genes with a two-fold change in expression were considered differentially regulated by lncRNA-*ROR*. The genes were mapped onto KEGG pathways using DAVID version 6.7 [38].

### Western blot

Cells were harvested at the indicated times and rinsed twice with PBS. Cell extracts were prepared with lysis buffer and centrifuged at 13,000 g for 30 min at 4 °C. Protein samples were separated by sodium dodecyl sulfate–polyacrylamide gel electrophoresis (SDS-PAGE) in 7.5 % (wt/vol) polyacrylamide gels and transferred to polyvinylidene fluoride membranes. After blocking with 5 % BSA for 1 h at room temperature, the membrane was incubated with 2.5 μg/mL antibody in 5 % BSA overnight at 4 °C. The membrane was then incubated with a secondary antibody conjugated to a fluorescent tag (Invitrogen). The band signals were visualized and quantified using the Odyssey Infrared Imagining System (LI-COR, USA). The following antibodies were used: Anti-TESC (Abcam, USA), Anti-H3K9me3 (Abcam, USA), and β-actin (Sigma-Aldrich).

### Immunohistochemical staining

For immunohistochemical staining, tissues sections were incubated at 4 °C overnight with a rat anti-human TESC antibody (Proteintech, USA) at a dilution of 1:100. Sections were then rinsed in PBS-T (PBS containing 0.05 % Triton X-100), and biotinylated anti-rat secondary antibody was then added at a 1:500 dilution at room temperature for 1 h. After washing twice with PBS-T, the slides were incubated with streptavidin-horseradish peroxidase (BD Biosciences, USA) and diaminobenzidine substrate for colorimetric development.

### Small interfering RNA

The knockdown of *TESC* was performed by using siRNA. Cells were seeded at 200,000 cells per well in 6-well plates and transfected using Lipofectamine 2000 (Invitrogen) in Opti-MEM I Reduced Serum Medium with 125 pmol siRNA (Invitrogen). At 48 h post-transfection, the cells were harvested in Trizol for RNA isolation (Invitrogen) or lysed in RIPA lysis buffer for western blotting.

### Cytoplasmic and nuclear RNA isolation

Cytoplasmic and nuclear RNA were extracted using Thermo Fisher BioReagents (Thermo Fisher, USA) according to the manufacturer’s instructions. RT-PCR was performed to amplify the localization of the *ROR* assay as follows: 1 μL 3 × Klen-Taq I Mix, 1 μL cDNA, and 0.5 μL each 10 μM primer were combined under liquid wax. After incubation at 95 °C for 2 min, the cDNA was amplified for 35 cycles at 95 °C for 30 s, optimal annealing temperature for 30 s, extension at 72 °C for 30 s, and a final extension at 72 °C for 5 min.

### DNA pull-down assay

Double-stranded DNA oligos were synthesized by PCR and labelled with biotin-14-dCTP according to the manufacturer’s instructions (Invitrogen). *In vitro* synthesized DNA was diluted in 10 mM Tris-HCl (pH 7.4), 25 mM NaCl, 10 mM MgCl2, and 10 % glycerol and incubated for 2 h with total RNA. Seventy five microliters of Dynabeads MyOne Streptavidin C1 beads (Invitrogen) were used to pull down the biotinylated DNA at room temperature for 25 min in 1 × binding and washing buffer (5 mM Tris-HCl 7.5, 0.5 mM EDTA, 1 M NaCl, 0.005 % Tween 20). The beads-DNA-RNAs were then washed with 1 × binding and washing buffer five times. The RNA was precipitated and diluted in 50 μL DEPC water followed by 2 min at 80 °C and 5 min at 65 °C. After the co-precipitated RNAs were isolated and treated with DNase I (New England BioLabs, USA), cDNA was synthesized using the PrimeScript RT reagent kit (Takara, Japan). RT-PCR was performing as follows: 1 μL of 3 × Klen-Taq I Mix, 1 μL cDNA, and 0.5 μL of each 10 μM primer were combined under liquid wax. After incubation at 95 °C for 2 min, the cDNA was amplified for 33 cycles at 95 °C for 30 s, optimal annealing temperature for 30 s, extension at 72 °C for 30 s, and a final extension at 72 °C for 5 min.

The TaqMan assay of the ABI 7500 Real-Time PCR Systems was performed to detect the quality of the *ROR* pulled down by Dynabeads MyOne Streptavidin C1 beads. Primers and probes labeled at their 5’ and 3’ ends with FAM and Black Hole Quencher-1 (BHQ-1) or Minor Groove Binder (MGB) were designed to target *ROR* and the negative control *KCNQ1OT1*. Probe and primer specificities were assessed *in silico* using the BLAST tools from the NCBI GenBank. The amplification reactions were optimized individually for all of the probes and associated primers. Each reaction was conducted in a total volume of 10 μL consisting of 0.6 μL 25 Mm MgCl_2,_ 0.25 μL 10 Mm dNTPs, 2 μL 5 × Q buffer, 0.25 μL of each 10 μM primer, 0.1 μL of TaqMan probe, 0.1 μL 5 U/μL Hotstar, 0.1 μL ROX dye reference, and 4 μL template. The assay thermal conditions were as follows: 60 °C for 1 min and 95 °C for 15 min followed by 45 cycles of 95 °C for 15 s and optimized annealing temperature for 1 min for each probe.

### Chromatin immunoprecipitation (ChIP)

ChIP assays were performed as previously described [39]. One hundred million cells were fixed with 1 % formaldehyde and sonicated for 8 min (10 s on and 15 s off) on ice with a 2 mm microtip at 40 % output control and 90 % duty cycle settings. The sonicated chromatin (0.5 mL) was clarified by centrifugation. To perform ChIP, sonicated chromatin (150 μL) was nine-fold diluted and protein G-agarose (60 μL) was added (Millipore), which was followed by 2 h of shaking at 4 C. This mixture was then briefly centrifuged at 1,000 rpm for 5 min, and the supernatant was collected into a new tube. KMT1C/G9a, dimethyl-H3-K27 (lysine 27 of histone H3) antibodies were obtained from Abcam (Abcam) and added to the supernatant overnight at 4 °C. PureProteome™ Protein A and Protein G Magnetic Beads (60 μL) (Millipore) were used to pull down the protein at 4 °C for 6 h. The DNA that was released from the bound chromatin after cross-linking reversal and proteinase K treatment was precipitated and diluted in 100 μL 0.2 M glycine.

The PCR conditions (3 μL under liquid wax) contained 2 μL ChIP (or input) DNA, 0.5 mM appropriate primer pairs, 50 μM deoxynucleotide triphosphate, and 0.2 U Klen-Taq I (Ab Peptides, USA). Standard PCR was performed using an ABI Prism 7500 Sequence Detection System (Applied Biosystems, USA) and the Power SYBR® Green PCR Master Mix (Applied Biosystems). Standard PCR conditions were as follows: 50 °C for 15 min and 94 °C for 2 min, which was followed by 40 cycles of 94 °C for 20 s, optimal annealing temperature at 30 s, extension at 72 °C for 35 s, and detection of fluorescence signal at 86 °C.

### Nuclear protein extraction

Cytoplasmic and nuclear proteins were extracted using Thermo Fisher BioReagents (Thermo Fisher) according to the manufacturer’s instructions.

### Production of purified RNA and protein *in vitro*

The DNA template for *ROR* synthesis was amplified by PCR, which contained an RNA polymerase T7 promoter site upstream of the sequence. DNA was isolated and purified using anion exchange columns (Qiagen) and sequenced to confirm that additional mutations had not been incorporated. *In vitro* RNA transcription synthesis used the mMESSAGE mMACHINE Kit according to the manufacturer’s instructions (Ambion, USA). RNA was purified by the MEGAclear Transcription Clean-Up Kit (Ambion).

The region encoding G9A was amplified by PCR from pBABE-FLAG-hG9a (Addgene, USA) and cloned into pET28a. Proteins were purified with nickel agarose (Thermo Fisher) and measured by the BCA method (BioRad, USA). The N-terminal FLAG-tag protein was expressed in 293 T and then purified with M2 flag beads (Sigma-Aldrich) and eluted with flag peptide (Sigma-Aldrich). Purified, concentrated proteins were stored at -20 °C in a buffer of 20 mM HEPES, pH 7.0, 100 mM NaCl, 0.5 mM EDTA, and 5 % glycerol.

### RNA-protein competition assay

The double-stranded DNA oligos were synthesized by PCR and labeled with Biotin-14-dCTP according to the manufacturer’s instructions (Invitrogen). *In vitro* synthesized DNA was mixed with 15 μg purified protein in 20 mM HEPES, pH 7.0, 100 mM NaCl, 5 % glycerol, 10 mM DTT, and 0.5 mM EDTA at room temperature for 30 min. Serial dilutions of the purified or total RNA prepared in the reaction buffer were added to the above DNA/protein hybrid at different ratios at room temperature for 30 min. Seventy-five microliters of Dynabeads MyOne Streptavidin C1 beads were used to pull down the biotinylated DNA at room temperature for 25 min in 1 × binding and washing buffer (5 mM Tris–HCl 7.5, 0.5 mM EDTA, 1 M NaCl, 0.005 % Tween 20). The beads were then washed with 1 × binding and washing buffer five times. The RNA was precipitated and diluted in 50 μL DEPC water and then maintained for 2 min at 80 °C and 5 min at 65 °C. After the co-precipitated RNAs were isolated and treated with DNase I (New England BioLabs, USA), cDNA was synthesized using the PrimeScript RT reagent kit (Takara). RT-PCR was performing as follows: 1 μL 3 × Klen-Taq I Mix, 1 μL cDNA, and 0.5 μL each 10 μM primers were combined under liquid wax. After incubation at 95 °C for 2 min, cDNA was amplified for 35 cycles at 95 °C for 30 s, optimal annealing temperature for 30 s and extension at 72 °C for 30 s, and final extension at 72 °C for 5 min. Protein-precipitated turbo DNase composed of 4 μL DNase was used to digest the complex for 30 min at 37 °C. Proteins were eluted with heating at 60 °C for 5 min in 1 × SDS PAGE loading buffer. The supernatant was then used for gel analysis.

### RNA immunoprecipitation (RIP)

One hundred million cells were harvested by trypsinization and resuspended in 2 mL PBS with RNA and protein inhibitors. The nuclei were pelleted by centrifugation at 2,500 G for 15 min, and the nuclear pellet was resuspended in 1 mL RIP buffer (150 mM KCl, 25 mM Tris pH 7.4, 5 MmEDTA, 0.5 mM DTT, 0.5 % NP40, 9 μg/mL leupeptin, 9 μg/mL pepstatin, 10 μg/mL chymostatin, 3 μg/mL aprotinin, 1 mM PMSF, and 100 U/mL RNA inhibitor). Resuspended nuclei were split into two fractions of 500 μL each and mechanically sheared using a sonicator at 40 % duty for 2.5 min. The nuclear membranes and debris were pelleted by centrifugation at 13,000 rpm for 10 min at 4 °C. To perform RIP, sonicated chromatin (150 μL) was four-fold diluted, and protein G-agarose (60 μL) was added and then shaken for 1 h at 4 °C and briefly centrifuged at 1,000 rpm for 5 min. The supernatant was then collected into new tubes, and KMT1C/G9a antibodies obtained from Abcam were added to the supernatant overnight at 4 °C. PureProteome™ Protein A and Protein G magnetic beads (60 μL) (Millipore, USA) were used to pull down the protein at 4 °C for 4 h. The RNA was precipitated and diluted in 50 μL 0.2 M glycine. The cDNA was synthesized using the PrimeScript RT reagent kit (Takara), and RT-PCR was performed under liquid wax in a reaction containing 2 μL RIP sample (or input) DNA, 0.5 mM appropriate primer pairs, 50 μM deoxynucleotide triphosphate, and 0.2 U Klen-Taq I (Ab Peptides, St. Louis, MO, USA). The positive control experiment was conducted in mouse fibroblast MBW2 cells. The MBW2 cells were cultured from an F1 newborn mouse derived from breeding a *Musspretu*s male with a C57B/6 female in our laboratory as previously described [6]. The primer used for amplifying mouse *Kcnq1ot1* is listed in Additional file 7: Table S3 (*Kcnq1ot1*-1 F, *Kcnq1ot1*-1R).

### Statistical analysis

All of the experiments were performed in triplicate, and the data were expressed as the mean ± standard deviation (SD). The comparative threshold cycle method was applied in the quantitative real-time RT-PCR assay according to the ΔΔ threshold cycle method.

## Additional files

Additional file 1: Figure S1. Validation of four siRNAs mediated knockdowns of *ROR* in AGS and HT29 cells. siROR-1 could efficiently silence *ROR* at the mRNA transcript level compared with that of the other three siRNAs in AGS and HT29 cells. All experiments were performed 48 h following siRNA (125 pmol) and control siRNA (125 pmol) administration. **P* <0.05: compared with the control and NC. NC: non-silencing control. Additional file 2: Table S1. Gene expression by genome-wide cDNA array in untreated tumor cells and *ROR* knockdown cells of AGS. (‘+’ upregulated, ‘-’ downregulated). Additional file 3: Figure S2. Tumor activity did not change after depletion of selected gene candidates. a-f. The effect of *ROR* silencing on *AKR1C3* (a), *AKR1C1* (b), *LMO4* (c), *MGST1* (d), *LXN* (e), and *TIMP3* (f) expression. Real-time PCR showing six remarkable downregulated genes after *ROR* depletion. All of the data are presented as the mean ± SD. **P* <0.05: compared with the control. g-, h. Transwell and soft agar assay showing no change in tumor activity after silencing of *AKR1C3*, *AKR1C1*, *LMO4*, *MGST1*, *LXN*, and *TIMP3*, respectively. Bars: 500 μm. Additional file 4: Table S2. Genes with a four-fold change after *ROR* depletion in HT29 cells by genome-wide cDNA array. (‘+’ upregulated, ‘-’ downregulated). Additional file 5: Figure S3. Histone H3K9 trimethylation detected by western blot after *ROR* silencing in tumor cells. Western blot demonstrated that the level of total H3K9 trimethylation was not changed in *ROR* silenced AGS and HT29 tumor cells. Additional file 6: Figure S4.
*ROR* lncRNA failed to bind with the G9A protein. a Schematic diagram of *ROR* lncRNA. S1 through S6: primer names; sites a, b, and c: different detecting locations of RNA IP. b RNA ChIP assay demonstrating the interaction of *ROR* and G9A in tumor cells. The positive control experiment was conducted in mouse fibroblast MBW2 cells and *Kcnq1ot1* was used as positive control for RNA IP. Negative control: control without antibody. Additional file 7: Table S3. Primers, siRNA, and shRNA sequences used in this study.
