# Erratum to: Long non-coding RNA ROR decoys gene-specific histone methylation to promote tumorigenesis

**DOI:** 10.1186/s13059-015-0852-5

**Published:** 2015-12-09

**Authors:** Jiayan Fan, Yue Xing, Xuyang Wen, Renbin Jia, Hongyan Ni, Jie He, Xia Ding, Hui Pan, Guanxiang Qian, Shengfang Ge, Andrew R. Hoffman, He Zhang, Xianqun Fan

**Affiliations:** Department of Ophthalmology, Ninth People’s Hospital, Shanghai JiaoTong University School of Medicine, Shanghai, 200025 P. R. China; Department of Biochemistry and Molecular Biology, Ninth People’s Hospital, Shanghai JiaoTong University School of Medicine, Shanghai, P. R. China; VA Palo Alto Health Care System, Palo Alto, CA 94304 USA

After the publication of this work [1], we noticed an error whereby two corresponding author’s names were missed from the Acknowledgements section. The corrected statement is provided below:
