# Supplementary figures and images for: Long non-coding RNA ROR decoys gene-specific histone methylation to promote tumorigenesis
Source: Genome Biol. 2015 Jul 14;16(1):139. doi: 10.1186/s13059-015-0705-2 (PMC4499915; doi:10.1186/s13059-015-0705-2)

**A**

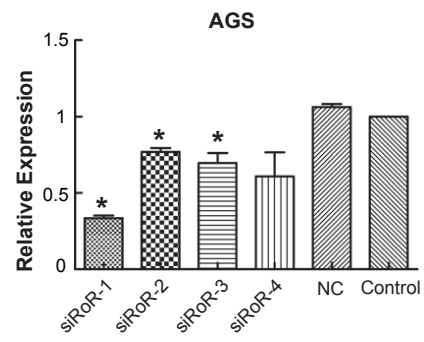

**B**

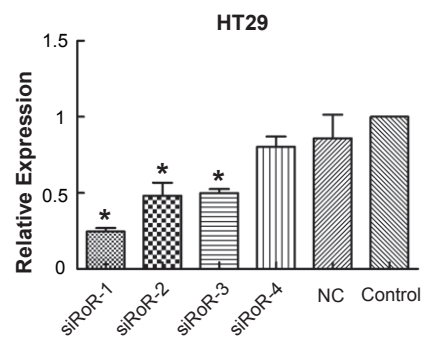

**Supplementary Figure 1 Silencing of *RoR* by siRNAs**

Supplement: Additional file 1: Figure S1. — Validation of four siRNAs mediated knockdowns of ROR in AGS and HT29 cells. siROR-1 could efficiently silence ROR at the mRNA transcript level compared with that of the other three siRNAs in AGS and HT29 cells. All experiments were performed 48 h following siRNA (125 pmol) and control siRNA (125 pmol) administration. *P <0.05: compared with the control and NC. NC: non-silencing control. [file 13059_2015_705_MOESM1_ESM.pdf]

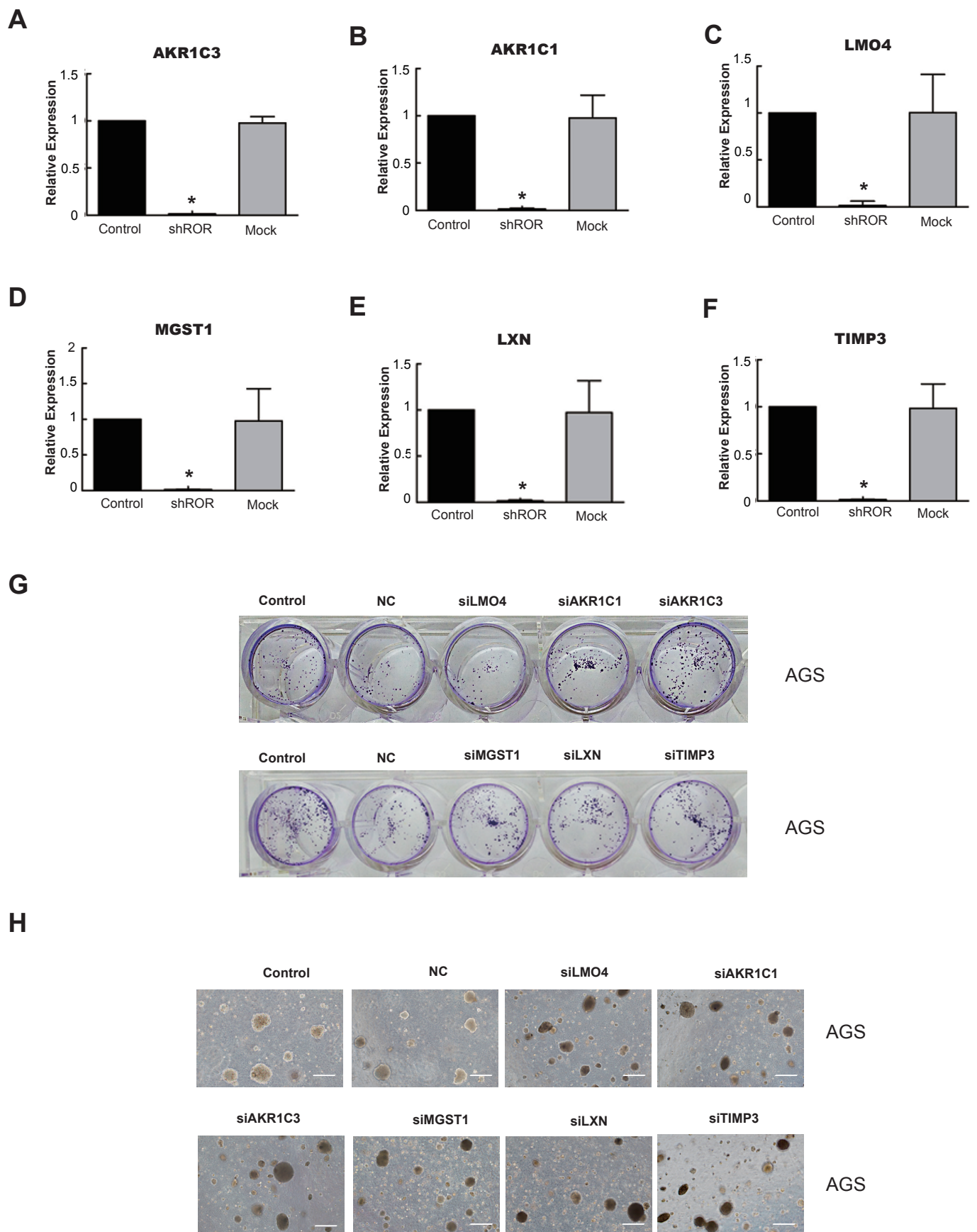

**Supplementary Figure 2 Selected altered genes in *RoR* silencing tumor cells**

Supplement: Additional file 3: Figure S2. — Tumor activity did not change after depletion of selected gene candidates. a-f. The effect of ROR silencing on AKR1C3 (a), AKR1C1 (b), LMO4 (c), MGST1 (d), LXN (e), and TIMP3 (f) expression. Real-time PCR showing six remarkable downregulated genes after ROR depletion. All of the data are presented as the mean ± SD. *P <0.05: compared with the control. g-, h. Transwell and soft agar assay showing no change in tumor activity after silencing of AKR1C3, AKR1C1, LMO4, MGST1, LXN, and TIMP3, respectively. Bars: 500 μm. [file 13059_2015_705_MOESM3_ESM.pdf]

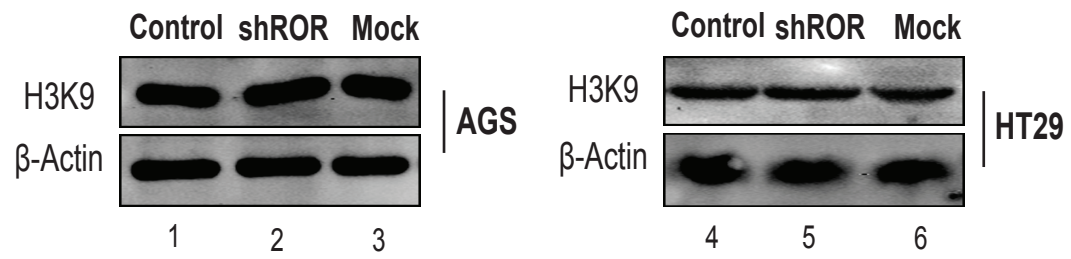

**Supplementary Figure 3 Histone H3K9 trimethylation detected by western blot**

Supplement: Additional file 5: Figure S3. — Histone H3K9 trimethylation detected by western blot after ROR silencing in tumor cells. Western blot demonstrated that the level of total H3K9 trimethylation was not changed in ROR silenced AGS and HT29 tumor cells. [file 13059_2015_705_MOESM5_ESM.pdf]

**A**

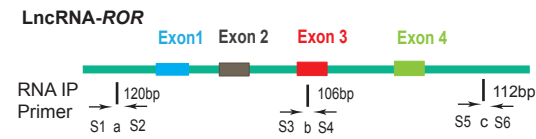

**B**

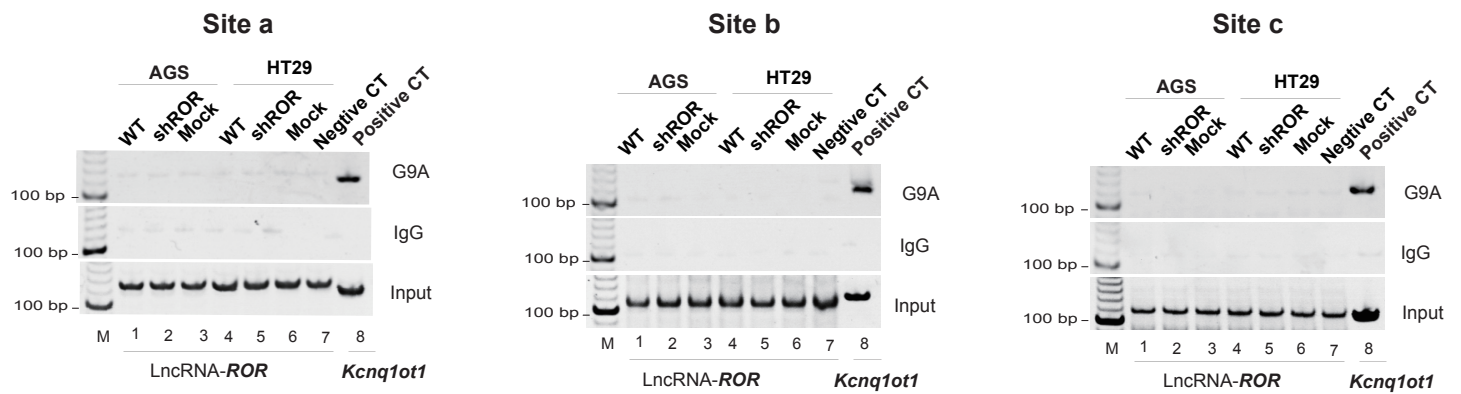

**Supplementary Figure 4 *ROR* Failed to bind with G9A protein**

Supplement: Additional file 6: Figure S4. — ROR lncRNA failed to bind with the G9A protein. a Schematic diagram of ROR lncRNA. S1 through S6: primer names; sites a, b, and c: different detecting locations of RNA IP. b RNA ChIP assay demonstrating the interaction of ROR and G9A in tumor cells. The positive control experiment was conducted in mouse fibroblast MBW2 cells and Kcnq1ot1 was used as positive control for RNA IP. Negative control: control without antibody. [file 13059_2015_705_MOESM6_ESM.pdf]
