# Supplementary material for: Long non-coding RNA ROR decoys gene-specific histone methylation to promote tumorigenesis
Source: Genome Biol. 2015 Jul 14;16(1):139. doi: 10.1186/s13059-015-0705-2 (PMC4499915; doi:10.1186/s13059-015-0705-2)
Supplement: Additional file 2: Table S1. — Gene expression by genome-wide cDNA array in untreated tumor cells and ROR knockdown cells of AGS. (‘+’ upregulated, ‘-’ downregulated). [file 13059_2015_705_MOESM2_ESM.docx]

**Tables S1** Gene expression by genome-wide cDNA array in untreated tumor cells and *ROR* knockdown cells of AGS. (‘+’ upregulated, ‘-’ downregulated)

| **Gene** | **Fold change ≥4** | **Gene** | **Fold change ≥4** |
| --- | --- | --- | --- |
| CXCL5 | -70.65648 | TSC22D3 | -5.582847 |
| AKR1C3 | -32.36079 | CCL20 | -5.536687 |
| TESC | -26.4389 | IQGAP2 | -5.529768 |
| PRKD3 | -21.52516 | ZIC2 | -5.465472 |
| CAMK2N1 | -17.4776 | TIMP3 | -5.420455 |
| AKR1C1 | -15.08998 | LXN | -5.034146 |
| MGST1 | -14.0819 | KLK7 | -4.984076 |
| AKR1B10 | -11.27271 | PPARG | -4.960907 |
| QPCT | -12.67064 | CKMT1A | -4.808394 |
| GNG12 | -12.66578 | DDIT3 | -4.76786 |
| G0S2 | -10.67999 | HTRA1 | -4.71383 |
| LMO4 | -10.00276 | SNTB1 | -4.610077 |
| AKR1C2 | -9.17512 | CEACAM5 | -4.344716 |
| UGT8 | -8.988672 | AKR1B15 | -4.269733 |
| SPRED1 | -8.859775 | LEF1 | -4.139791 |
| NUPR1 | -8.530108 | PRSS1 | -4.130914 |
| ABCG1 | -7.927083 | HOPX | +8.8777987 |
| NEO1 | -7.847831 | CAV1 | +7.9436772 |
| IGF2 | -6.194836 | KCNJ8 | +7.1927278 |
| PHGR1 | -7.597359 | FLRT3 | +5.8818378 |
| BHLHE41 | -7.081382 | SPICE1 | +5.785942 |
| C2orf55 | -6.633871 | CD24 | +5.2748644 |
| LCN2 | -6.294911 | ITGB8 | +4.9813922 |
| ST6GALNAC1 | -6.054698 | ODAM | +4.6255984 |
| GJB2 | -5.958553 | DKK1 | +4.5453047 |
| DDIT4 | -5.708106 | SCIN | +4.4487037 |
| LYZ | -5.706824 | PRKACB | +4.2393351 |
| GALC | -5.646426 | SPTSSB | +4.1847661 |
| TMEM45B | -5.618091 | TFPI | +4.1689642 |
