# Supplementary material for: Long non-coding RNA ROR decoys gene-specific histone methylation to promote tumorigenesis
Source: Genome Biol. 2015 Jul 14;16(1):139. doi: 10.1186/s13059-015-0705-2 (PMC4499915; doi:10.1186/s13059-015-0705-2)
Supplement: Additional file 4: Table S2. — Genes with a four-fold change after ROR depletion in HT29 cells by genome-wide cDNA array. (‘+’ upregulated, ‘-’ downregulated). [file 13059_2015_705_MOESM4_ESM.docx]

**Table S2** Genes with a four-fold change after *ROR* depletion in HT29 cells by genome-wide cDNA array. (‘+’ upregulated, ‘-’ downregulated)

| **Gene** | **Fold change ≥4** | **Gene** | **Fold change ≥4** |
| --- | --- | --- | --- |
| CXCR4 | -27.00203 | KIAA1199 | -4.773754 |
| CLEC3A | -23.57062 | FGFR2 | -4.761513 |
| NNMT | -14.6224 | PLXND1 | -4.706316 |
| DPYSL2 | -11.88989 | LINC00514 | -4.654303 |
| DNAH2 | -11.79329 | LXN | -4.646981 |
| TGM2 | -11.05626 | SERPINA5 | -4.644578 |
| EGR1 | -10.44034 | GALNT12 | -4.618144 |
| NOX1 | -10.13211 | CCDC104 | -4.596881 |
| TGFBI | -9.907128 | TCTN1 | -4.567272 |
| ADAMTS6 | -9.514585 | CYP2B6 | -4.522356 |
| GBP2 | -8.537362 | CLRN3 | -4.515882 |
| GJB1 | -8.437327 | NR0B2 | -4.510703 |
| FOS | -8.179485 | LIMS2 | -4.481526 |
| CLGN | -7.706911 | ANXA9 | -4.480619 |
| C14orf105 | -7.445374 | CAPN12 | -4.435376 |
| IFITM1 | -7.44127 | SLC44A3 | -4.418744 |
| ALDH1A1 | -7.372634 | ADD3 | -4.407287 |
| TESC | -7.356584 | SEC16B | -4.398849 |
| AGR3 | -7.316709 | SERPINA1 | -4.398116 |
| PBX1 | -7.250459 | HBEGF | -4.346046 |
| FRY | -7.126536 | LARP6 | -4.343541 |
| LAMP3 | -7.005826 | LYPD6B | -4.285067 |
| SH3BGRL | -6.713566 | LIME1 | -4.271158 |
| MYLK | -6.530417 | RABGAP1L | -4.247644 |
| FAM129A | -6.384126 | TSPAN6 | -4.231419 |
| SLC5A1 | -6.202969 | GATA2 | -4.205783 |
| TBXAS1 | -6.193582 | XKRX | -4.190524 |
| NUPR1 | -6.137976 | PDE9A | -4.171345 |
| XAF1 | -6.095688 | CA12 | -4.166789 |
| CEACAM1 | -5.96231 | B3GNT1 | -4.113414 |
| ITGB8 | -5.941175 | PION | -4.091246 |
| FABP6 | -5.889085 | TDGF1 | -4.079902 |
| TRIM22 | -5.847903 | QPRT | -4.060668 |
| PLEKHB1 | -5.807413 | ITPR2 | -4.049783 |
| PDK4 | -5.769727 | CFTR | -4.030652 |
| KLK11 | -5.649746 | ARL6 | -4.019621 |
| TNFSF10 | -5.586169 | AKNA | -4.007934 |
| CPVL | -5.558858 | SPRR1B | +34.89749 |
| PAPSS2 | -5.505838 | DHRS9 | +31.86573 |
| PDZK1 | -5.458864 | ARL4C | +11.4287 |
| SLC12A2 | -5.346343 | SPRR1 | +8.764828 |
| KCNH2 | -5.305728 | ETS1 | +8.021921 |
| ERBB3 | -5.287043 | HBB | +7.872315 |
| TEAD2 | -5.196055 | GEM | +7.79071 |
| COL9A3 | -5.18501 | ZNF185 | +6.656715 |
| RABL5 | -5.181794 | SFT2D3 | +5.744185 |
| IDH1 | -5.170672 | HMGA2 | +5.495513 |
| PITX2 | -5.13109 | GULP1 | +5.05378 |
| PECR | -5.110508 | EMP1 | +4.873781 |
| ITGAM | -5.065054 | ASPH | +4.826925 |
| CSPG4 | -5.06369 | HIST1H4 | +4.781603 |
| IGF2 | -5.029772 | DST | +4.617368 |
| DEPTOR | -5.009059 | DLEU2 | +4.589602 |
| NFE2L3 | -5.006292 | ZNF529 | +4.534023 |
| CAT | -4.958623 | DKK1 | +4.296474 |
| UGTA | -4.942946 | RAD18 | +4.296089 |
| MAP2K6 | -4.942002 | DEPDC7 | +4.29036 |
| AQP5 | -4.915222 | ZNF611 | +4.233148 |
| MMP13 | -4.896672 | STX1A | +4.057153 |
| VNN1 | -4.868559 | IL1RN | +4.032416 |
| SERPINA3 | -4.863349 | HCFC2 | +4.021086 |
| CMPK2 | -4.83906 | C1orf109 | +4.015867 |
| FILIP1L | -4.803314 |  |  |
