# Supplementary material for: Long non-coding RNA ROR decoys gene-specific histone methylation to promote tumorigenesis
Source: Genome Biol. 2015 Jul 14;16(1):139. doi: 10.1186/s13059-015-0705-2 (PMC4499915; doi:10.1186/s13059-015-0705-2)
Supplement: Additional file 7: Table S3. — Primers, siRNA, and shRNA sequences used in this study. [file 13059_2015_705_MOESM7_ESM.docx]

**Table S3**. Primers, siRNA, and shRNA sequences used in this study.

| **Primer name** | **Sequences (5’-3’)** | **Purpose** | **Temperature, cycle** |
| --- | --- | --- | --- |
| TESC-F | TGTCGGGAAACCCTCACATCGAG | RT-PCR | 65 °C, 33 cycles |
| TESC-R | AGGAAGCGGACGTGCATCTTGG | RT-PCR |  |
| ROR-F | CATTTTCCATCCTGCTGTTCAGAGT | RT-PCR | 65 °C, 33 cycles |
| ROR-R | GGCTCTTTCTCTCCTGTGGTTTCAT | RT-PCR |  |
| TESC-T1 | AGGTAAAGACATCTCAAAAGGCCAATC | ChIP | 60 °C, 33 cycles |
| TESC-T2 | GGCCTAAATTTCGCTAAGACGC | ChIP |  |
| TESC-T3 | GAAGGGACGAGGTACGTGAGCA | ChIP | 65°C, 33 cycles |
| TESC-T4 | GAGCGGGGCCTCATATAACGG | ChIP |  |
| TESC-T5 | ACCTGAGAGTCGTTTGCATTCGTGA | ChIP | 65 °C, 33 cycles |
| TESC-T6 | GCTGCCTGAACCCATTTTACAGATG | ChIP |  |
| TESC-T7 | TTCATGCTCGCTGACTTCCTTGAC | Biotinylated TESC-1 | 60 °C, 33 cycles |
| TESC-T8 | GGCCTCATATAACGGCGGA | Biotinylated TESC-1 |  |
| TESC-T9 | TCCTGCAGTTTGAGGTGCCTC | Biotinylated TESC-2 | 65 °C, 33 cycles |
| TESC-T10 | TGCAGCAGTGCTCACGTACCT | Biotinylated TESC-2 |  |
| TESC-T11 | AGGTGGAGGTTGCAGTGAGCT | Biotinylated TESC-3 | 62 °C, 33 cycles |
| TESC-T12 | GTAATTCTGGACCCATTACAGGAATG | Biotinylated TESC-3 |  |
| ROR-S1 | TGATCCACTGGTCAGATCCCAGGTC | RIP | 65 °C, 33 cycles |
| ROR-S2 | CTGCACAATGGCACTGCAGCACT | RIP |  |
| ROR-S3 | CATTTTCCATCCTGCTGTTCAGAGT | RIP | 65 °C, 33 cycles |
| ROR-S4 | GGCTCTTTCTCTCCTGTGGTTTCAT | RIP |  |
| ROR-S5 | CTCATCTCCTGCACAGACAGAGAGCA | RIP | 65 °C, 33 cycles |
| ROR-S6 | AGGGTTCAAGAACAACCAGCTGTGGC | RIP |  |
| Kcnq1ot1-1F | TCGGTGGGCTTCTCCTCGGC | RIP | 65 °C, 33 cycles |
| Kcnq1ot1-1R | CGATCACACGGATGAAAACCACGC | RIP |  |
| KCNQ1OT1-2F | CCACTTAGCAAGCCAGGACTA | DNA Pull Down | 65 °C, 33 cycles |
| KCNQ1OT1-2R | GACATCCTTGGCCTACTCCTTA | DNA Pull Down |  |
| ROR-sh1-F | TTGACAGTGAGCGCGCCTGAGAGTTGGCATGA  ATATAGTGAAGCCACAGATGTA | shRNA Constructs |  |
| ROR-sh1-R | TCCGAGGCAGTAGGCAAGCCTGAGAGTTGG  CATGAATATACATCTGTGGCTTCA | shRNA Constructs |  |
| ROR-sh2-F | TTGACAGTGAGCGCGGGTTAAAGACACAGGGGA  AATAGTGAAGCCACAGATGTA | shRNA Constructs |  |
| ROR-sh2-R | TCCGAGGCAGTAGGCAAGGGTTAAAGACACAGG  GGAAATACATCTGTGGCTTCA | shRNA Constructs |  |
| siROR-1 | CCTGAGAGTTGGCATGAAT | siROR |  |
| siROR-2 | GGTTAAAGACACAGGGGAA | siROR |  |
| siROR-3 | CTTCAGGATAAATGAGAAA | siROR |  |
| siROR-4 | GGAGAGGAAGCCTGAGAGT | siROR |  |
| siTESC-1 | GAAACCCTCACATCGAGAA | siTESC |  |
| siTESC-2 | CCGCTTCCTTAACATCCAA | siTESC |  |
| siTESC | CTTGTAGGGTATGGTATGT | siTESC |  |
| siRNA-NC | ATCCACTACCGTTGTTATAGGTG | Negative siRNA |  |
| ROR-T7-F | TAATACGACTCACTATAGGTTTTATTTTTTGAGGAACT | *In vitro* RNA transcription syntheses | 65 °C |
| ROR-T7-R | GGTGAAATAAACAGCCATGTTGCTCAC | *In vitro* RNA transcription syntheses |  |
| ROR-Ftaq | GATGGCATTGTCGCTAAGTAAGAA | qPCR Taqman | 65 °C |
| ROR-Rtaq | TTTCATTGTCCTGGGGTGTTC | qPCR Taqman |  |
| ROR-Probe | FAM- AGTATGGATGTGATGAGAGAC-MGB | qPCR Taqman |  |
| KCNQ1OT1-Ftaq | ACACCACTTAGCAAGCCAGGA | qPCR Taqman | 65 °C |
| KCNQ1OT1-Rtaq | GACCATCAACCCTATGAGCTACAC | qPCR Taqman |  |
| KCNQ1OT1-probe | FAM-CCAAGGATGTCATCCTGGCCATACCC- BHQ | qPCR Taqman |  |
